# Optimised emollient mixture for skin barrier repair: Applications to global child health

**DOI:** 10.7189/jogh.12.03019

**Published:** 2022-04-30

**Authors:** Peter M Elias, Mao-Qiang Man, Gary L Darmstadt

**Affiliations:** 1 Department of Dermatology, University of California, Northern California Institute for Research and Education, and Veterans Affairs Health Care Center, San Francisco, California, USA; 2 Prematurity Research Center, Department of Pediatrics, Stanford University School of Medicine, Stanford, California, USA

## INTRODUCTION: IMPORTANCE OF SKIN BARRIER HOMEOSTASIS

Maintenance of epidermal permeability barrier homeostasis is required for life in a desiccating, often hostile, terrestrial environment. Children over six months of age and adults who are armed with robust metabolic machinery readily cope with most external stressors [1]. In contrast, individuals with sub-optimal permeability barriers – including the aged, children and adults with atopic spectrum disorders, and preterm and low birth weight infants – respond poorly to the same external challenges [2]. These individuals display not only suboptimal permeability barrier function that can lead to substantial caloric loss and flawed cutaneous antimicrobial defense, but also reduced thresholds to irritant and allergic inflammatory stimuli [3]. Defective permeability barrier function increases energy expenditures in preterm infants due to caloric losses associated with high rates of transepidermal water loss [4]. Moreover, children and adults in low-resource settings are at particularly increased risks for all these complications, in part due to their marginal nutritional status, and they are likewise at increased risks for sepsis due to bacterial invasion through breaches in skin barrier defense in contexts of high environmental pathogenic load [5,6].

## INEFFECTIVE TO HARMFUL EFFECTS OF COMMON SKIN CARE PRODUCTS

The cosmetic and pharmaceutical industries have been quick to respond, generating vast numbers of products that claim often-unproven benefits for the skin. Recent research has shown that, although nearly all of these products (including those commonly used for newborn infants in sub-Saharan Africa and South Asia) can improve skin hydration (moisturisation), they can also harm the permeability barriers of those individuals afflicted with flawed barriers, including neonates, the aged, patients with atopic spectrum disorders or acne rosacea, and others with self-characterised “sensitive skin” [7,8]. While these formulations may provide temporary relief from dryness and perhaps itching, they typically demand ever-more frequent applications due to their negative impact on the permeability barrier. Such a vicious circle potentially benefits the manufacturer far more than the user. Thus, while widespread use of skin applications for newborn infants and children throughout South Asia, sub-Saharan Africa and the Mediterranean region provides a platform for scaling up skin care interventions through public and private delivery [9-12], current practices may do more harm than good.

**Figure:**
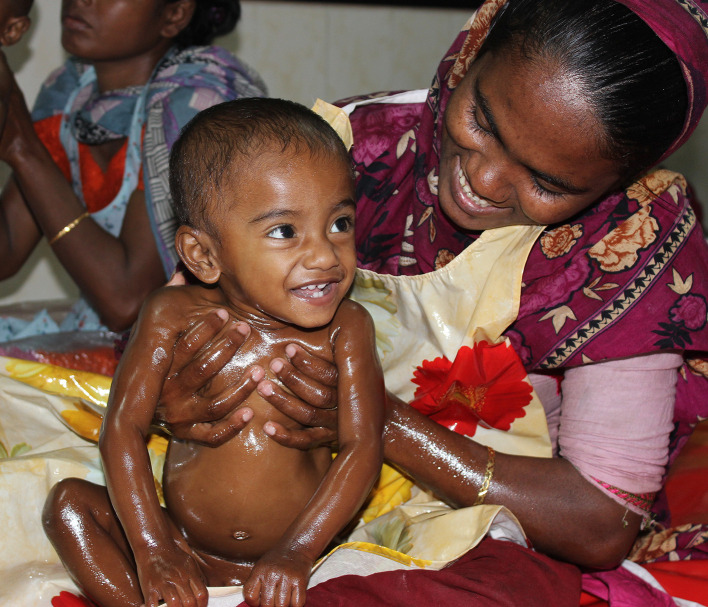
Photo: Mother and young child enjoying emollient therapy at icddr,b in Dhaka, Bangladesh. From Dr KM Shahunja with permission and caregiver consent.

## USE OF EMOLLIENTS TO IMPROVE SKIN BARRIER FUNCTION AND HEALTH

Applications of some moisturisers, such as Aquaphor and Eucerin, have been shown to enhance skin barrier function, but not clinical outcomes in preterm neonates [13]. In contrast, several studies in cohorts of preterm infants in low-and middle-income countries have shown that sunflower seed oil (SSO) with high linoleic acid content (eg, >60%) provides benefits for the skin barrier and the survival, health, growth, and development of newborn infants [14-17]. Moreover, a recent randomised controlled trial in young children at 2-24 months of age with severe acute malnutrition in Bangladesh showed that topical applications of SSO improved barrier function, while reducing nosocomial infection risk and inflammatory markers (eg, C-reactive protein) in age-ranked subgroups of children [18]. Because topical fatty acids (including linoleic acid, an essential fatty acid) are absorbed into the blood, there was a trend toward a higher rate of weight gain in the SSO-treated children compared to the control group [18,19]. Topical triglyceride-enriched preparations exploit a panoply of endogenous lipases, which in turn generate their constituent free fatty acids, including linoleic acid.

Emollient therapy with SSO also significantly impacts skin microbiome composition, specifically increasing microbial diversity [20]. Notably, a reduction in such microbial diversity can be a harbinger of atopic dermatitis in affected infants [21]. It should also be noted that some products, intended to enhance the permeability barrier, also contain ingredients that up-regulate antimicrobial defense, particularly the expression of the cathelicidin protein, LL-37. It is becoming increasingly clear that these two critical defensive functions (ie, permeability barrier homeostasis and antimicrobial defense) are integrated and co-regulated [22].

While highly promising as therapeutic agents, natural vegetable oils such as SSO are also variable in content and quality, depending on conditions of production, processing, and storage [8]. Lack of quality control translates into the potential for unreliable skin barrier and health effects across time and geography.

## TOWARD OPTIMISED SKIN BARRIER FUNCTION AND HEALTH

Based on considerations of skin biology, a subclass of barrier repair formulations has been developed in an attempt to mimic the mixture of three physiologic lipids (ceramides, cholesterol, and free fatty acids) that are responsible for cutaneous permeability barrier function. Formulations that contain an equimolar distribution of these three lipids are not harmful to disturbed skin, but incomplete or imbalanced mixtures aggravate underlying barrier defects, presumably by destabilising the functional properties of the skin’s lipid-based barrier (**Table 1**). SSO, for example, is not balanced in its lipid composition, and use of neat oil has the potential to retard skin barrier maturation [23]. Subsequent studies identified what has been termed an optimal molar ratio (ie, 3:1:1 mixtures) of these lipids, which accelerates barrier recovery after cutaneous insults ranging from solvent applications, detergent treatment, or cellophane tape stripping (as used in mouse models) [24]. A ceramide-dominant, yet balanced product, developed on the basis of these principles, marketed widely as EpiCeram® emulsion (Primus Pharmaceuticals, Scottsdale, Arizona), has demonstrated remarkable efficacy in pediatric patients with atopic dermatitis [25,26]. In these studies, enhancement of the permeability barrier was accompanied by improved skin integrity and cohesion (ie, resistance to tape stripping), and simultaneously improved antimicrobial defense by stimulating expression of the epidermal antimicrobial peptides, LL-37 and human beta-defensin2 (hBD2) [22]. Once again, enhanced permeability barrier function was paralleled by augmented antimicrobial defense.

**Table 1:** Impact of moisturisers and barrier repair formulations on barrier homeostasis

Petrolatum	Immediate; incomplete; short-term repair
Glycerol	Hydration; building block for glyerolipid synthesis
Moisturisers	Short-term hydration; destabilises barrier
Optimised emollient mixture	Accelerates barrier recovery; boosts innate immunity; is anti-inflammatory
Physiologic lipids:	
Single species	Inhibits barrier recovery
Dual species	Inhibits barrier recovery
Triple species (equimolar)	Allows normal recovery
Triple species (optimal molar)	Accelerates barrier repair; boosts innate immunity; is anti-inflammatory

**Figure 1:**
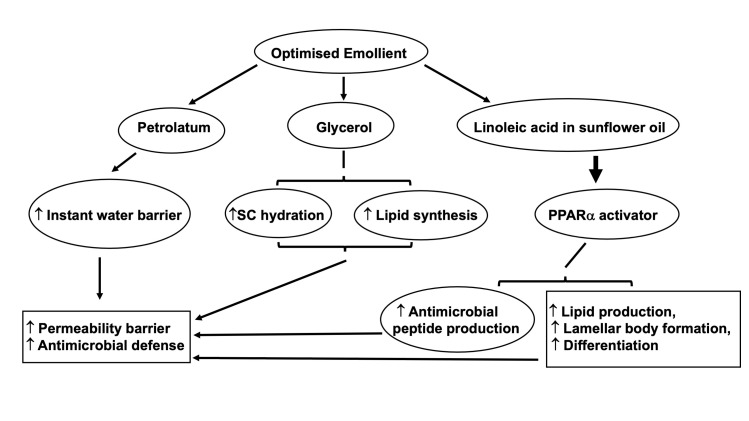
The putative mechanisms by which a topical optimised mixture improves permeability barrier homeostasis. In the mixture, petrolatum and lanolin instantly improve the permeability barrier, while glycerol improves barrier (stratum corneum, SC) hydration and accelerates permeability barrier repair. Linoleic acid in both sunflower oil and borage oils activates peroxisome proliferator-activated receptors (PPAR), resulting in increased production of lipids and antimicrobial peptides, and stimulation of lamellar body secretion and membrane maturation. Consequently, both permeability barrier and antimicrobial barriers are improved. Adapted from reference [27].

Yet, the efficacy of EpiCeram is dependent upon its composition of three key lipids at high final concentrations of over 5%, and the costs of synthetic ceramides and cholesterol are substantial, resulting in a product that, though highly effective, is prohibitively expensive for deployment in low-resource contexts. With the assistance of a grant from the Bill and Melinda Gates Foundation, we developed a readily manufactured, cosmetically acceptable formulation of inexpensive ingredients, at a critical ratio, optimal concentrations, and reduced pH, designed for twice-daily application, that provided benefits comparable or superior to EpiCeram [27]. A key component of this Optimized Emollient Mixture (OEM) is SSO, which the senior author has deployed with both barrier restorative and nutritional benefits in low- and middle-income countries [5,6,8,14-20]. The other ingredients provide additional benefits for epidermal structure and function (**Figure 1**). Lowering the pH of the product serves to further up-regulate antimicrobial peptide production, critical for cutaneous innate immunity [28]. Reducing surface pH also accelerates barrier maturation, which is particularly important in improving skin barrier function in young infants. Moreover, maintaining a low surface pH (ie, below 5) attenuates the development of atopic dermatitis induced by repeated hapten challenges, and prevents the subsequent progression of atopic dermatitis to asthma in murine models [28].

Using EpiCeram as the key comparator, we have found that the OEM improved permeability barrier homeostasis with even greater efficacy, while enhancing epidermal antimicrobial peptide expression still further (see **Figure 1** Panel B in reference [27]). In preclinical studies, the OEM also attenuated the development of inflammatory skin abnormalities in a mouse model of atopic dermatitis, when the full concentration of ingredients was applied twice daily for 10 days (unpublished data). Finally, in a small cohort of patients (n = 7) with moderate-to-severe atopic dermatitis, the use of OEM twice daily for 5-7 days improved clinical features within five days of topical treatment (unpublished data).

In separate studies, we have identified further benefits of barrier restorative therapy in aged populations. In chronologically aged skin, epidermal production of the cytokines, TNF-alpha, IL-1-beta, and IL-6, increase, and these cytokines ultimately elevate circulating levels of these biomarkers [29]. Conversely, restoration of normal barrier function with barrier repair formulations lowered circulating levels of these cytokines in aged mice and humans, indicating a reduction in systemic immune activation and inflammation.

Together, these findings suggest that the deployment of the OEM in low- and middle-income country settings could provide benefits across the life course, for premature neonates, as well as for elderly adults. OEM therapy may be important for accelerating recovery of young children from severe acute malnutrition, and it seems plausible that the OEM could prevent inflammation in young children at risk for developing atopic dermatitis and prevent or delay the subsequent progression of atopic dermatitis to mucosal atopy, including asthma, allergic rhinitis, and food allergies.

## CONCLUSION

As we turn our attention to promoting survival, health, and well-being in the era of the Sustainable Development Goals, and as we embrace the imperative to move beyond “Survive” to “Thrive and Transform”, greater consideration is needed to optimizing skin care and skin barrier function and health, especially in the most vulnerable segments of populations worldwide.
